# Decreased influenza activity during the COVID-19 pandemic in Ghana, 2020

**DOI:** 10.3389/fpubh.2023.1290553

**Published:** 2024-01-16

**Authors:** Ivy Asantewaa Asante, Stephen Ofori Nyarko, Yaw Awuku-Larbi, Richard Asomadu Obeng, Gifty Mawuli Sarpong, Esinam Aku Apefa Amenuvor, Mildred Adusei-Poku, Linda Boatemaa, Vanessa Magnusen, Jennifer Wutsika, Samuel Ago, Lorreta Kwasah, Juliet Wordui, Roberta Aprilyn Tackie, Dennis Odai Laryea, Franklin Asiedu-Bekoe, William Asiedu, Daniel Lartei Mingle, Edward Owusu Nyarko, Anne Fox, Shirley C. Nimo-Paintsil, Naiki Attram, Terrel Sanders, William Kwabena Ampofo

**Affiliations:** ^1^National Influenza Centre, Noguchi Memorial Institute for Medical Research, University of Ghana, Accra, Ghana; ^2^U.S. Naval Medical Research Unit EURAFCENT, Accra, Ghana; ^3^Department of Medical Microbiology, University of Ghana Medical School, University of Ghana, Accra, Ghana; ^4^West African Centre for Cell Biology of Infectious Pathogens, University of Ghana, Accra, Ghana; ^5^Ghana Health Service, Ministry of Health, Accra, Ghana; ^6^Public Health Division, 37 Military Hospital, Ghana Armed Forces, Accra, Ghana

**Keywords:** influenza activity, COVID-19, pandemic, surveillance, Ghana

## Abstract

**Introduction:**

The COVID-19 pandemic had a significant effect on influenza activity globally. In this study, we analyzed trends of influenza activity in 2020 during the COVID-19 pandemic in Ghana.

**Methods:**

This was a cross-sectional study using active prospective influenza surveillance data from 29 sentinel sites. At the sentinel sites, we enrolled patients presenting with symptoms based on the WHO case definition for influenza-like illness (ILI) and severe acute respiratory illness (SARI). Oro and nasopharyngeal swabs were collected from patients and tested for the presence of influenza viruses using specific primers and probes described by the US-CDC. The percentage of positivity for influenza between 2017–2019 and 2021 was compared to 2020. Using the test for proportions in STATA 17.0 we estimated the difference in influenza activities between two periods.

**Results and discussion:**

Influenza activity occurred in a single wave during the 2020 surveillance season into 2021, September 28 2020–March 7 2021 (week 40, 2020–week 9, 2021). Influenza activity in 2020 was significantly lower compared to previous years (2017– 2019, 2021). Influenza A (H3) was more commonly detected during the early part of the year (December 30, 2019–March 8, 2020), while influenza B Victoria was more commonly detected toward the end of the year (September 28–December 28). In Ghana, adherence to the community mitigation strategies introduced to reduce transmission of SARS-CoV-2, which affected the transmission of other infectious diseases, may have also impacted the transmission of influenza. To the best of our knowledge, this is the first study in Ghana to describe the effect of the COVID-19 pandemic on influenza activity. The continuation and strict adherence to the non-pharmaceutical interventions at the community level can help reduce influenza transmission in subsequent seasons.

## Introduction

The first cases of the novel coronavirus, Severe Acute Respiratory Syndrome Coronavirus-2 (SARS-CoV-2), were reported in Ghana on March 12, 2020 (1). Since then, cases increased steadily and as of May 21, 2023, the country had recorded 171, 740 cases and 1,462 deaths from SARS-CoV-2 (2). Since the first recorded cases, the country instituted several non-pharmaceutical measures to curb the spread of the infection. These included encouraging the population to wear face masks, wash hands frequently using soap under running water, maintain a distance of approximately 2 meters among people, ban social gatherings, and close up cinemas and places of leisure where people congregated for a long time. These interventions significantly contributed to slowing down the spread of infection throughout the country.

Surveillance for influenza viruses among the general population in Ghana was established in 2007 (3), when outbreaks of highly pathogenic avian influenza viruses led to the death and culling of >100,000 poultry (4). This surveillance system has contributed significantly to providing data on circulating influenza virus strains in the country. Data from Ghana are shared with the World Health Organization (WHO) Global Influenza Surveillance and Response System (GISRS) network. Samples are also shared with a WHO Collaborating Centre on influenza viruses for investigations contributing to the annual WHO influenza vaccine formulations. Ghana has subsequently been designated a National Influenza Centre by the WHO with support from the Ministry of Health (MoH).

It has been suggested that measures established to curb the spread of SARS-CoV-2 may have contributed to a general reduction in the spread of infectious diseases throughout the country. However, this has not been demonstrated yet for any infectious disease in Ghana. In this study, we analyzed trends of influenza activity during the COVID-19 pandemic in Ghana.

## Methodology

**Figure 1:**
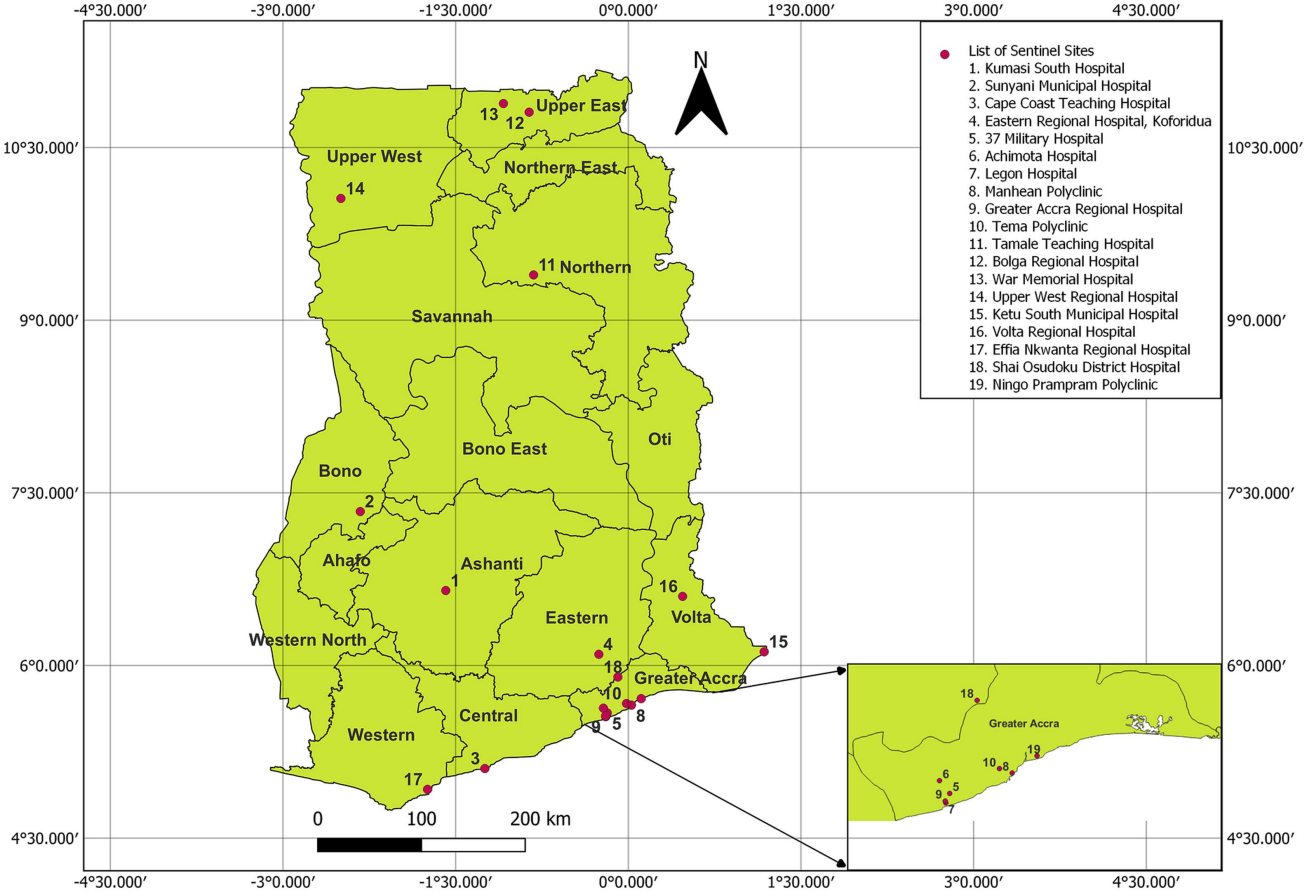
Map of Ghana showing regions with influenza sentinel sites. Regions are written in bold, while sentinel sites are displayed in numbers and red dots. Source: NIC Ghana. Note: Only 19/29 sentinel sites are indicated. Those located on Ghana Armed Forces military sites, with the exception of 37 Military Hospital, are not depicted for security reasons. The map was created using QGIS version 3.26.1-Buenos Aires with the Ghana boundary coordinates obtained from Ghana Statistical Service (GSS).

We conducted active prospective influenza surveillance from 29 sentinel sites established by the Ghana Health Service (GHS) of the MoH and the Ghana Armed Forces throughout the country (see Figure 1). These sentinel sites correspond to regional hospitals, clinics, and Medical Reception Stations of the Ghana Armed Forces. We enrolled patients presenting with symptoms based on the WHO case definition for influenza-like illness (ILI) and severe acute respiratory illness (SARI), which include history or measured fever of ≥38°C and cough with onset within the last 10 days (ILI), and requiring hospitalization for SARI cases (5). In addition, we collected data from enrolled patients, including demographic characteristics, clinical presentation, and underlying clinical conditions. Respiratory samples (nasopharyngeal and oropharyngeal swabs for laboratory testing of influenza) from patients were collected into viral transport medium (VTM) and transported to the National Influenza Centre (NIC) located at the Noguchi Memorial Institute for Medical Research (NMIMR) at 4–8°C for testing. Samples were processed for the presence of influenza viruses using specific primers, probes, and assays designed by the US Centers for Disease Control and Prevention (CDC). The NIC utilizes multiplex influenza and SARS-CoV-2 primer/probe set from the CDC in recent times.

We compared influenza activity in 2020 to previous seasons (2017–2019, 2021). The number of samples tested and the percentage of respiratory samples received at the NIC that tested positive for influenza for each year were mapped out by epidemiological weeks to assess influenza activity. The positivity percent for influenza for 2017–2019 and 2021 was compared to 2020 using the *z*-test for proportions in STATA 17.0, to estimate the difference in influenza activities between periods and the logit method used to compute the limit of confidence intervals. We also defined a wave as a consecutive increase in influenza activity over two or more consecutive weeks with a corresponding consecutive decrease for two or more weeks.

### Ethics statement

This epidemiologic activity was approved by the Naval Medical Research Command Institutional Review Board, and the Office of Research Administration as public health surveillance. The Ghana Health Service also concurred that this protocol is exempt from ethical considerations because it falls under public health surveillance. The sentinel sites for routine national influenza virus surveillance in Ghana were set up in collaboration with the Ghana Health Service, as well as the Ghana Armed Forces, as part of the Integrated Disease Surveillance and Response (IDSR) system of the Ghana Health Service, which is the health delivery division within the Ministry of Health. Due to this, oral consent was sought from participants before sample collection. All procedures were performed according to relevant guidelines and regulations. No administrative permissions were required to access this data. Data is kept on a password-protected computer and backed up daily. Data was anonymized before use. Only laboratory identities were used during data analysis.

## Results

In 2020, the NIC processed a total of 2,550 samples from 29 sentinel sites. Of these samples, we processed samples from more males (54%) than females. The demographic details of samples analyzed in 2020 are shown in Table 1. Influenza affected the younger population (0–14 years) more frequently than any other age group in Ghana. Influenza B Victoria lineage viruses were the dominant strains circulating for the year, together with influenza A (H3).

**Table 1:** Demographic characteristics of influenza cases received and processed at the Ghana NIC, 2020.

*N* = 2,550					
Variables	No. of samples processed	Influenza positives	Influenza A subtypes		Influenza B lineage
			Influenza A[H1N1] pdm09	Influenza AH3	Influenza B Victoria
		*n* (%)	*n* (%)	*n* (%)	*n* (%)
Age group, (Mean ± SD)	(26.80 ± 0.38)				
0–4 years	315	15(4.76)	2 (13.33)	6 (40.00)	7 (46.67)
5–14 years	199	13 (6.53)	–	10 (76.92)	3 (23.08)
15–24 years	553	16 (2.89)	1 (6.25)	4 (25.00)	11 (68.75)
25–44 years	839	19 (2.26)	2 (10.53)	5 (26.32)	12 (63.16)
45–64 years	258	2 (0.78)	–	2 (100.00)	–
>64 years	81	3 (3.70)	1 (33.33)	2 (66.67)	–
Missing values*	305	9 (2.96)	–	5 (55.56)	4 (44.44)
Sex					
Male	1,382	42 (3)	4 (10)	17 (40)	21 (50)
Female	1,141	35 (3)	2 (5.72)	17 (48.57)	16 (45.71)
Missing values	27	–	–	–	–
Total	2,550	77 (3.02)	6 (7.79)	34 (44.16)	37 (48.05)

*The missing values in the table are due to the incomplete data for selected COVID-19 samples tested for the presence of influenza viruses.

Influenza activity in 2020 was significantly lower compared to previous years (2017–2019). The comparison of influenza percent positivity showed 3.0% (77/2550, 95% confidence interval [CI] = 2.4–3.8%) of samples testing positive in 2020 vs. 10.38% (438/4022, 95% CI = 9.9–11.9%) in 2021 [value of *p*<0.001], 22.4% (1,181/5265, 95% CI = 21.3–23.6%) in 2019 [value of *p*<0.001], 14.7% (741/5072, 95% CI = 13.7–15.7%) in 2018 [value of *p*<0.001], and 9.2% (469/5075, 95% CI = 8.4–10.1%) in 2017 [value of *p*<0.001].

**Figure 2:**
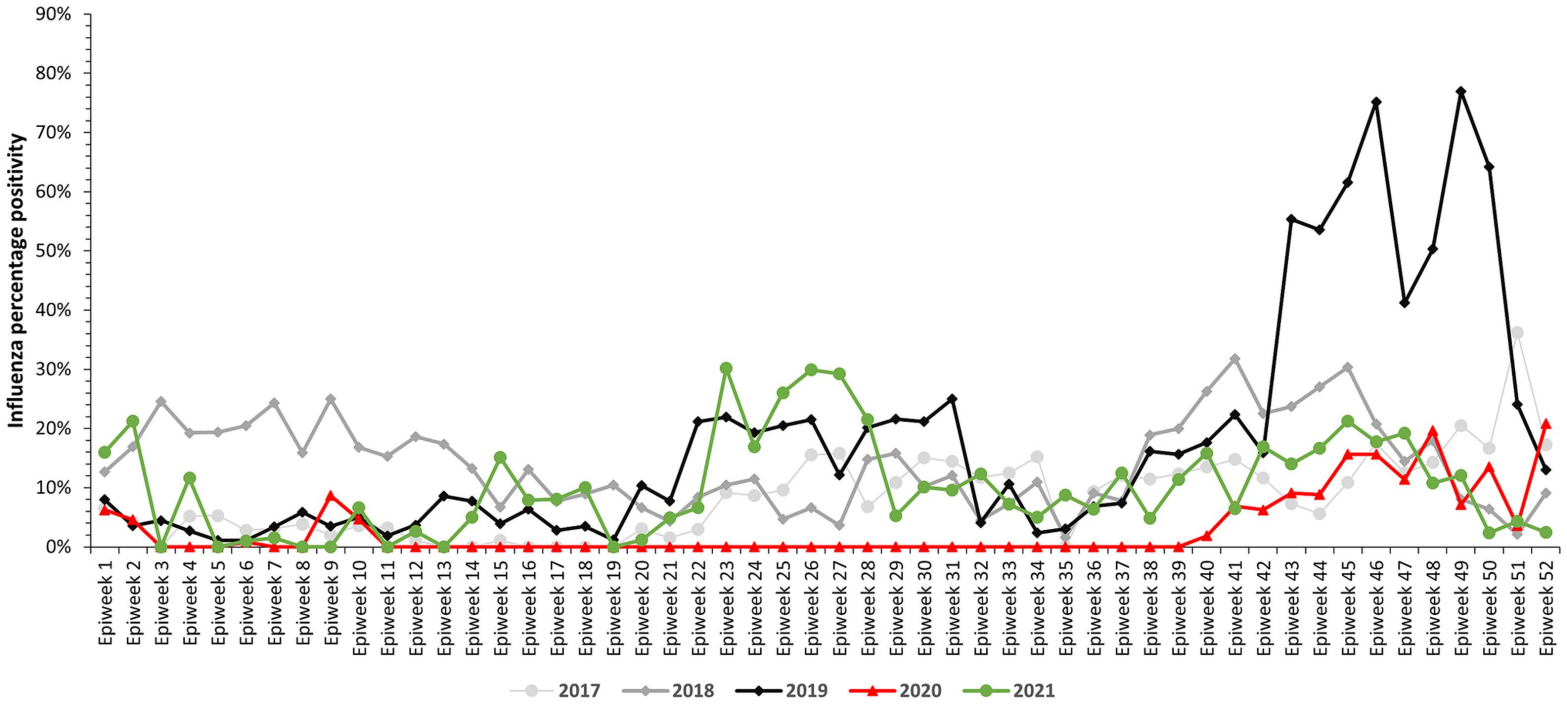
Percentage testing positive for influenza by week and year – Ghana influenza surveillance, 2017–2021.

**Figure 3:**
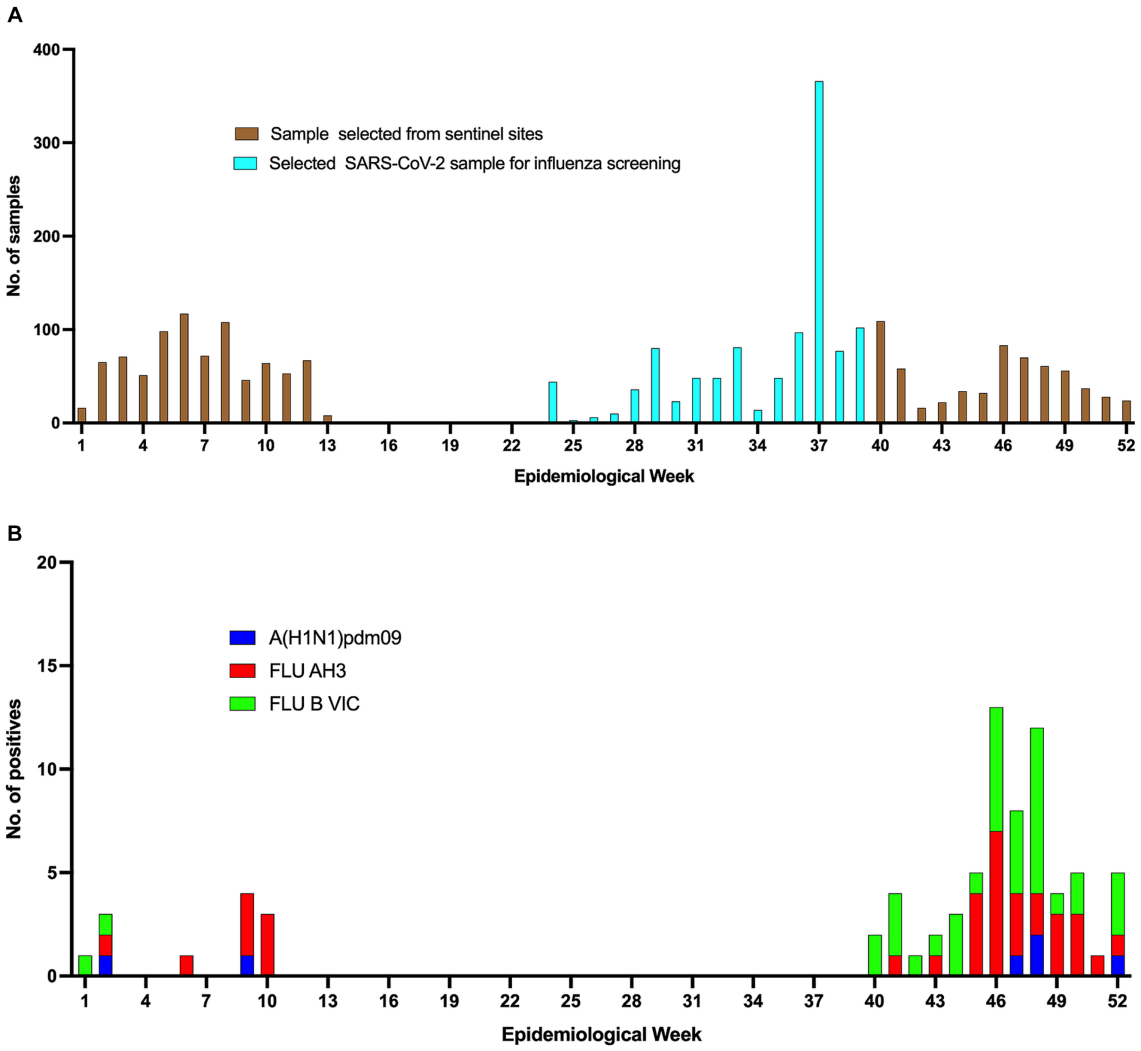
Samples received at the NIC, Ghana and the positives obtained. **(A)** The cyan bars represent samples screened for influenza from the daily COVID-19 samples received for testing, while the brown bars represent samples received at the NIC for routine influenza testing. **(B)** Shows the number and subtypes of influenza positives for the various epi-weeks.

Influenza activity occurred in a single wave in the 2020 surveillance season continuing into 2021, September 282,020 to March07 2021 (week 40, 2020 to week 9) (see Figure 2). In 2020, influenza positivity percentage peaked in weeks 48 (November 23–29) and 52 (December 21–27) at 20 and 21%, respectively, during the wave (see Figure 2). During this period, influenza B Victoria was detected in 54% of the samples that tested positive for influenza (see Figure 3B). The average influenza positivity during the wave showed 10.3% (65/630, 95% CI = 8.1–13.0%) in 2020 vs. 11.3% (103/911, 95% CI = 9.3–13.5%) in 2021 [*p* = 0.536], 48.3% (817/1692, 95% CI = 45.9–50.7%) in 2019 [value of *p*<0.001], 19.3% (223/1156, 95% CI = 17.0–21.6%) in 2018 [value of *p*<0.001], and 16% (225/1411, 95% CI = 14.0–17.9%) in 2017 [value of *p*<0.001].

In the early period of 2020; from December 30, 2019 to March 8, 2020 (epidemiological weeks 1–10), influenza was detected in weeks 1–2 (December 30, 2019–January 12, 2020), 6 (February 3–9, 2020) and 9–10 (February 24–March 8, 2020) (Figure 2). During this period, the highest influenza positivity percentage was recorded from late February to early March (week 9) at 9%. During the same period, influenza A (H3) was more commonly detected among the samples that tested positive for influenza (66.7% positive for A (H3) vs. 16.7% positive for A[H1N1]pdm09 and influenza B Victoria, value of *p* = 0.011) (see Figure 3B). The average influenza positivity during this period in 2020 was lower compared to previous years (2017–2019). The influenza positivity during this period (from week 1–10) showed 1.7% [12/708](95% CI = 0.7–2.6%) in 2020 vs. 3.7% [30/817] (95% CI = 2.4–5.0%) in 2019 [value of *p* = 0.019], 19.7% [228/1159](95% CI = 17.4–22.0%) in 2018 [value of *p*<0.001], and 3.6% [30/826] (95% CI = 2.4–4.9%) in 2017 [value of *p* = 0.021].

In 2020, sentinel sites submitted samples to the NIC from weeks 1–13 (December 30, 2019–March 29, 2020) and 40–52 (September 28 2020–December 28 2020) (Figure 3A). The median weekly number of samples tested for influenza decreased from 64 in weeks 1–13 to 37 in weeks 40–52, representing a percentage decrease of 42.1%. In addition, no influenza activity was reported from week 11–week 39. Between weeks 14–24, no samples were received by the NIC from the sentinel sites. The NIC, however, analysed 1,083 samples for COVID-19 diagnosis received by NMIMR from weeks 25–39 (a weekly average of 72 samples) for the presence of influenza viruses. All these 1,083 samples tested negative for influenza (see Figure 3A).

## Discussion

In response to the rising cases of SARS-CoV-2 in Ghana, the government of Ghana, in collaboration with the Ministry of Health and Ghana Health Service, introduced several non-pharmaceutical interventions to curb the spread of this virus. These interventions contributed to a reduction in the transmission of influenza in Ghana. Therefore, this paper sought to describe influenza activity (defined as influenza positivity rate among analyzed samples) during 2020.

The NIC is responsible for the virological surveillance of influenza activity in Ghana. Analysis of our 2020 data shows that influenza activity declined compared to previous years (2017–2019). Our findings correlate with the global influenza circulation attributable to the similar COVID-19 control measures were introduced across the globe, thus also affecting influenza transmission (6, 7). A Japanese study reported that the adherence to NPIs such as strict adherence to proper hand hygiene practices as well as international travel restrictions was associated with decreased influenza activity (8). A modeling study in Ghana showed that introduction and adherence of NPIs (such as use of face masks, hand washing, closure of international and land borders) as well as reduced mobility negatively impacted COVID-19 transmission in the early phase of the pandemic. However, the effect varied over time as the pandemic progressed (9).

While both influenza and SARS-CoV-2 spread primarily by aerosol transmission, data shows SARS-CoV-2 is generally more transmissible (R_0_ ranging from 1.8–3.21), (10, 11) than seasonal influenza (R_0_ ranging from 1.19–1.37) (12). Thus, potentially resulting in COVID-19 control measures having a greater impact on influenza than COVID-19. In addition, most clinical diagnoses with respiratory symptoms were preferentially tested for SARS-CoV-2 neglecting influenza, thus, contributing to the decline in the detection rates of influenza. Although there is an already established effective influenza surveillance system by the NIC-Ghana, this was interrupted during weeks 14–39 of the influenza season due to the relocation of resources and personnel for the detection of COVID-19. As a result, this negatively affected the monitoring of influenza activity.

In Ghana, influenza circulates all year-round with expected multiple peaks during the major (epi-weeks 14–26) and minor (epi-weeks 32–43) raining seasons (13). Our observation for 2020 was close to the expected characteristic seasonality in Ghana during the minor raining season. Despite the low influenza activity in 2020, there was an observed increase in influenza activity between weeks 40–52 when the sentinel sites started resending samples to the NIC for influenza testing. The increase may have been associated with the ease of restrictions on international travels in Ghana [announced on 1^st^ September, 2020, during epi-week 36] and the reopening of junior and senior high schools [returned on 5^th^ October, 2020, during epi-week 41] (14, 15). It has been reported that children and young adults are significant drivers for influenza transmission (16). Around the same period, the re-emergence of influenza activity was recorded in some parts of Asia (17), suggesting the likely resurgence of global influenza activity. In addition, studies have shown that international travelers contribute immensely to the transmission of the seasonal influenza virus globally (18). Influenza A (H3) and B Victoria dominated in the 2020 surveillance season. The influenza season in Ghana is driven by varying strains of seasonal influenza viruses (13). The transition between influenza strains and other respiratory pathogens remains unpredictable (16). Other countries like the US, attributed the rebound of influenza and other respiratory pathogens during the COVID-19 pandemic in 2020 to reopening of schools and offices (16). The resurgence of influenza activity may also be associated with reduced immunity.

The findings from the study showed a limitation in the likelihood of missing influenza cases between epi-weeks 13–24 due to samples not received from the sentinel sites. In addition, although samples received for SARS-CoV-2 testing were also tested for influenza, these samples were from individuals with clinical manifestations similar to COVID-19, thus less likely to detect influenza. Also, the low influenza detection should be interpreted with caution due to self-protecting behaviors of individuals in accessing healthcare during the pandemic. It is entirely possible that individuals with mild influenza illnesses preferred staying home instead of accessing healthcare, and therefore not detected by the influenza surveillance system.

This is the first study in Ghana to describe the effect of the COVID-19 pandemic on influenza activity. The continuation and strict adherence to the non-pharmaceutical interventions at the community level can help reduce influenza transmission in subsequent seasons.

## Data availability statement

The raw data supporting the conclusions of this article will be made available by the authors, without undue reservation.

## Ethics statement

The study involving humans was approved by the Naval Medical Research Command Institutional Review Board – Office of Research Administration (NAMRU3-PJT-21-01) as public health surveillance. The Ghana Health Service concurred that this protocol is exempt from ethical considerations including the informed consent process because it falls under public health surveillance. The sentinel sites for routine national influenza virus surveillance in Ghana were set up in collaboration with the Ghana Health Service as well as the Ghana Armed Forces as part of the Integrated Disease Surveillance and Response (IDSR) system of the Ghana Health Service, which is the health delivery division within the Ministry of Health. All procedures were performed according to relevant guidelines and regulations.

## Author contributions

IA: Conceptualization, Writing – original draft. SN: Data curation, Formal analysis, Writing – original draft. YA-L: Data curation, Formal analysis, Writing – original draft. RO: Conceptualization, Data curation, Formal analysis, Writing – original draft. GS: Writing – review & editing, Methodology. EA: Data curation, Writing – review & editing, Methodology. MA-P: Writing – review & editing, Methodology. LB: Writing – review & editing, Methodology. VM: Writing – review & editing, Methodology. JWu: Methodology, Writing – review & editing. SA: Methodology, Writing – review & editing. LK: Writing – review & editing, Methodology. JWo: Writing – review & editing, Methodology. RT: Writing – review & editing, Methodology. DL: Writing – review & editing. FA-B: Writing – review & editing. WAs: Writing – review & editing. DM: Writing – review & editing. EN: Writing – review & editing. AF: Writing – review & editing. SN-P: Writing – review & editing. NA: Writing – review & editing. TS: Writing – review & editing. WAm: Conceptualization, Supervision, Writing – review & editing.

## Author disclaimer

Some authors are military service members or employees of the U.S. Government (TS, SN-P and NA). This work was prepared as part of their official duties. Title 17, U.S.C. §105 provides that copyright protection under this title is not available for any work of the U.S. Government. Title 17, U.S.C. §101 defines a U.S. Government work as work prepared by a military service member or employee of the U.S. Government as part of that person’s official duties. The views expressed in this article are those of the authors and do not necessarily reflect the official policy or position of the Department of the Navy, Department of Defense, nor the U.S. Government.
