# Corrigendum: Decreased influenza activity during the COVID-19 pandemic in Ghana, 2020

**DOI:** 10.3389/fpubh.2024.1386351

**Published:** 2024-03-04

**Authors:** Ivy Asantewaa Asante, Stephen Ofori Nyarko, Yaw Awuku-Larbi, Richard Asomadu Obeng, Gifty Mawuli Sarpong, Esinam Aku Apefa Amenuvor, Mildred Adusei-Poku, Linda Boatemaa, Vanessa Magnusen, Jennifer Wutsika, Samuel Ago, Lorreta Kwasah, Juliet Wordui, Roberta Aprilyn Tackie, Dennis Odai Laryea, Franklin Asiedu-Bekoe, William Asiedu, Daniel Lartei Mingle, Edward Owusu Nyarko, Anne Fox, Shirley C. Nimo-Paintsil, Naiki Attram, Terrel Sanders, William Kwabena Ampofo

**Affiliations:** ^1^National Influenza Centre, Noguchi Memorial Institute for Medical Research, University of Ghana, Accra, Ghana; ^2^U.S. Naval Medical Research Unit EURAFCENT, Accra, Ghana; ^3^Department of Medical Microbiology, University of Ghana Medical School, University of Ghana, Accra, Ghana; ^4^West African Centre for Cell Biology of Infectious Pathogens, University of Ghana, Accra, Ghana; ^5^Ghana Health Service, Ministry of Health, Accra, Ghana; ^6^Public Health Division, 37 Military Hospital, Ghana Armed Forces, Accra, Ghana

**Keywords:** influenza activity, COVID-19, pandemic, surveillance, Ghana

In the section, Methodology, subheading, *Ethics statement*, the first sentence was incorrect.

This sentence previously stated:

“This epidemiologic activity was approved by the Noguchi Memorial Institute for Medical Research Institutional Review Board, the Naval Medical Research Command Institutional Review Board, and the Office of Research Administration as public health surveillance.”

The correct sentence appears below:

“This epidemiologic activity was approved by the Naval Medical Research Command Institutional Review Board, and the Office of Research Administration as public health surveillance.”

The Ethics statement was incorrect.

This text previously stated:

“The study involving humans was approved by the College of Health Sciences Ethical and Protocol Review Committee (CHS-Et/M.3-P 2.7/2017-2018), Noguchi Memorial Institute for Medical Research Institutional Review Board (NMIMR-IRB CPN 062/17-18), and the Naval Medical Research Command Institutional Review Board - Office of Research Administration (NAMRU3-PJT-21-01) as public health surveillance. The Ghana Health Service waived the requirement of written informed consent from participants or the participants' legal guardians/next of kin because this protocol falls under public health surveillance.”

The correct text appears below:

“The study involving humans was approved by the Naval Medical Research Command Institutional Review Board – Office of Research Administration (NAMRU3-PJT-21-01) as public health surveillance. The Ghana Health Service concurred that this protocol is exempt from ethical considerations including the informed consent process because it falls under public health surveillance. The sentinel sites for routine national influenza virus surveillance in Ghana were set up in collaboration with the Ghana Health Service as well as the Ghana Armed Forces as part of the Integrated Disease Surveillance and Response (IDSR) system of the Ghana Health Service, which is the health delivery division within the Ministry of Health. All procedures were performed according to relevant guidelines and regulations.”

The authors apologize for these errors and state that this does not change the scientific conclusions of the article in any way. The original article has been updated.

